# Synergistic and compensatory effects of two point mutations conferring target-site resistance to fipronil in the insect GABA receptor RDL

**DOI:** 10.1038/srep32335

**Published:** 2016-08-25

**Authors:** Yixi Zhang, Xiangkun Meng, Yuanxue Yang, Hong Li, Xin Wang, Baojun Yang, Jianhua Zhang, Chunrui Li, Neil S. Millar, Zewen Liu

**Affiliations:** 1 Key Laboratory of Integrated Management of Crop Diseases and Pests (Ministry of Education), College of Plant Protection, Nanjing Agricultural University, Weigang 1, Nanjing 210095, China; 2 Rice Technology Research and Development Center, China National Rice Research Institute, Hangzhou 310006, China; 3 Department of Neuroscience, Physiology & Pharmacology, University College London, London, WC1E 6BT, UK

## Abstract

Insecticide resistance can arise from a variety of mechanisms, including changes to the target site, but is often associated with substantial fitness costs to insects. Here we describe two resistance-associated target-site mutations that have synergistic and compensatory effects that combine to produce high and persistent levels of resistance to fipronil, an insecticide targeting on γ-aminobytyric acid (GABA) receptors. In *Nilaparvata lugens*, a major pest of rice crops in many parts of Asia, we have identified a single point mutation (A302S) in the GABA receptor RDL that has been identified previously in other species and which confers low levels of resistance to fipronil (23-fold) in *N. lugans*. In addition, we have identified a second resistance-associated RDL mutation (R300Q) that, in combination with A302S, is associated with much higher levels of resistance (237-fold). The R300Q mutation has not been detected in the absence of A302S in either laboratory-selected or field populations, presumably due to the high fitness cost associated with this mutation. Significantly, it appears that the A302S mutation is able to compensate for deleterious effects of R300Q mutation on fitness cost. These findings identify a novel resistance mechanism and may have important implications for the spread of insecticide resistance.

The use of chemical insecticides to control insect pests is extremely widespread, particularly in applications such as crop protection and animal health^1^. In addition to concerns about potential toxicity to non-target species, the extensive use of insecticides has been associated with the development of resistance within insect populations^2^,^3^. In addition to mechanisms of resistance that are associated with enhanced detoxification (metabolic resistance), resistance can also occur as a consequence of mutations within the insecticide target site (target-site resistance). However, an important feature of mutations conferring insecticide resistance is that they can also result in an increased fitness cost to the insect, a feature that can limit the spread of mutations within insect populations^4^.

There is evidence that mutations conferring high levels of insecticide resistance are often associated with the reduced insect survived to adulthood^5^, lower mating success^6^, increased susceptibility to natural enemies^7^,^8^, or greater mortality during over-wintering^8^. In rare cases, insects have developed mechanisms to overcome the disadvantages associated with insecticide resistance and, as a consequence, otherwise deleterious mutations have become established in insect populations. For example, fitness costs associated with the knock-down resistance (kdr) mutation in the insect sodium channel can be compensated for by mutations in acetylcholinesterase^5^. In *Culex* and *Anopheles* mosquito, duplication of the acetylcholinesterase gene can compensate for fitness costs, by a resistance-associated mutation in one gene copy being compensated for by the duplicated wild-type copy^9^,^10^,^11^.

Inhibitory γ-aminobutyric acid (GABA)-gated chloride channels are a diverse family of pentameric neurotransmitter receptors in both vertebrate and invertebrate species^12^. In insects, they form part of a family of ligand-gated chloride channels that include receptors for GABA, glutamate and histamine^13^ and are important targets for insecticides^14^. In common with other members of this family of neurotransmitter-gated ion channels, five transmembrane subunits co-assemble to form a central ion channel pore, with the agonist binding sites located at subunit interfaces in the extracellular domain. Each subunit in the pentameric receptor is a single polypeptide chain containing four α-helical transmembrane domains (TM1-TM4) with the second TM domain (TM2) from each subunit lining the ion-channel pore. There is evidence that several noncompetitive antagonists, such as fipronil, bind in the GABA receptor transmembrane pore domain and interact with amino acids located on TM2^3^,^15^,^16^. The first insect GABA-gated ion channel to be characterized in detail was RDL^17^, originally identified in *Drosophila* as being encoded by a gene associated with resistance to the insecticide dieldrin (*rdl*) ‘resistance to dieldrin’^18^,^19^. In addition to being the target site for dieldrin, the insect RDL receptor is a target for the widely used and commercially important insecticide fipronil^20^. Early studies demonstrated that resistance to insecticides such as dieldrin and fipronil were a consequence of a single point mutation (A302S), located within the TM2 domain of RDL^19^,^21^,^22^,^23^, that confers resistance to fipronil and is now widespread in field populations of several important insect pests.

Here we describe studies examining insecticide resistance mechanisms in *Nilaparvata lugens* (brown planthopper), a major pest of rice crops throughout Asia. Studies with both field populations and laboratory-selected insects have identified a second resistance-associated point mutation (R300Q) in fipronil-resistant *N. lugens*. The mutation R300Q is located two amino acids upstream of A302 in the TM2 domain of RDL. The present study with susceptible and resistant insects, combined with electrophysiological studies of wild-type and mutated RDL receptors, reveals that the R300Q mutation (in the absence of A302S) has a profoundly deleterious effect on the normal functioning of the RDL receptor and, as a consequence of this, it appears to be associated with a very high fitness cost. However, R300Q is able to act synergistically with A302S to confer substantially higher levels of resistance to fipronil than that are seen with A302S alone and with relatively low fitness cost. Our findings demonstrate an important compensatory mechanism, whereby the existence of one mutation (A302S) is able to compensate for the otherwise deleterious effects of a resistance-conferring mutation (R300Q) located within the same gene. It is a phenomenon that is likely to have important implications for the development and spread of resistance to fipronil.

## Results

### Fipronil resistance selection and mutation detection

**Figure 1:**
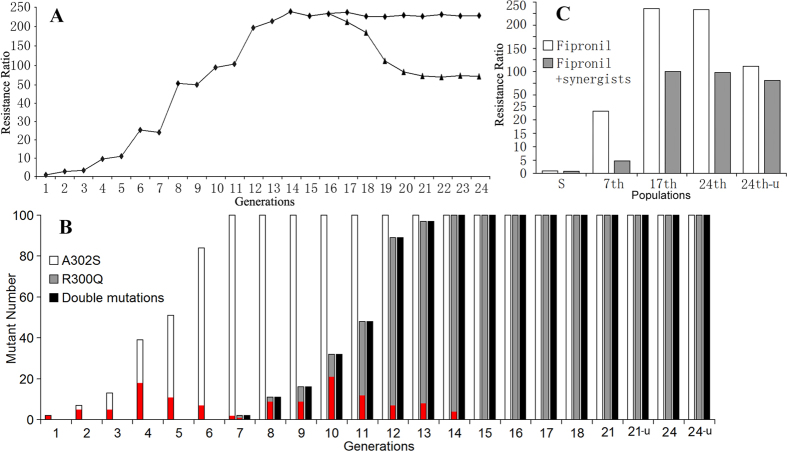
Laboratory selection and characterization of fipronil resistant *Nilaparvata lugens*. (**A**) The profile of fipronil resistance during laboratory selection of a field population of *N. lugens* (isolated from Chainat, Thailand) is illustrated. The graph illustrates changes in resistance ratios in subsequent generations during continuous selection with fipronil (diamonds) and after removal of fipronil selection after the 16th generation (triangles). (**B**) Mutation detection in insects collected during each generation of fipronil selection, illustrating the frequency of A302S and R300Q mutations. In each generation, 100 individuals were examined by nucleotide sequencing of the TM1-TM3 domain of RDL. In each column, the number of heterozygotes for either A302S or R300Q is indicated by a red bar. The frequency of double mutations (individuals containing both the A302S and R300Q mutation) is also illustrated. (**C**) The effects of synergists on fipronil resistance ratios are illustrated in insect populations isolated during various generations of selection.

A field population of *N. lugens* isolated from Chainat, Thailand was subjected to laboratory selection with fipronil. Resistance to fipronil developed rapidly and reached a peak level at the 14th generation, with a resistance ratio of 237-fold (Fig. 1A). Further selection after the 14th generation did not result in a further increase in fipronil resistance, with levels remaining close to 230-fold in subsequent generations. At the 16th generation, some insect samples were separated out to create a new population that was maintained without selection. In these un-selected generations (designated as ‘u’ in Fig. 1B,C), the resistance ratios decreased gradually and also reached a plateau, with levels close to 105-fold (Fig. 1A).

**Figure 2:**
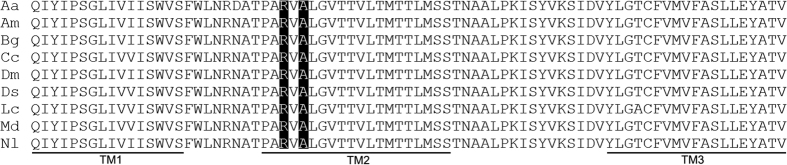
Alignment of RDL amino acid sequences from transmembrane domains 1 to 3 (TM1 to TM3) from various insect species. The positions of arginine (R) and alanine (A) amino acids, corresponding to the R300Q and A302S mutations, are indicated by a black background. Three transmembrane regions (TM1, TM2 and TM3) are underlined. Database accession numbers for the sequences are: *Aedes aegypti* (Aa), AAA68961; *Apis mellifera* (Am), AAC63381; *Blattella germanica* (Bg), AAB33733; *Ceratitis capitata* (Cc), AAD51101; *Drosophila melanogaster* (Dm), P25123; *Drosophila simulans* (Ds), AAK00512; *Lucilia cuprina* (Lc), AAB81966; *Musca domestica* (Md), AAC23602; *Nilaparvata lugens* (Nl), AGK30293.

Nucleotide sequencing was performed on 100 insects from each generation of fipronil selection. Sequencing of the transmembrane region of the GABA receptor RDL subunit identified an A302S mutation that has been described previously in several insect species (Fig. 2). The frequency of the A302S mutation within the population increased during early rounds of selection and reached 100% at the 7th generation (Fig. 1B). Another mutation, R300Q (two residues before A302S) was detected only after the 7th generation of selection. The frequency of the R300Q mutation reached 100% at the 14th generation of selection. An interesting finding was that, in every generation, the R300Q mutation was detected only in individuals that also contained the A302S mutation in RDL. After removal of fipronil selection after the 16th generation, the double mutation (R300Q/A302S) persisted at a frequency of 100% in the non-selected population.

Possible resistance mechanisms were examined by investigating the effect of synergists (Fig. 1C). In the 7th generation, in which all individuals contained the A302S mutation, synergists showed significant effects on fipronil toxicity and lowered the resistance ratios (RR) from 23.2-fold to 4.7-fold. This finding suggests that resistance in this population might largely be a consequence of metabolic mechanisms such as enhanced detoxification, rather than a consequence of the A302S mutation directly conferring target-site resistance to fipronil. In the 17th and 24th generations, in which all insects contained the double mutations (R300Q and A302S), synergists also had significant effects. After treatment with synergists, the RR remained above 96-fold, suggesting that both target insensitivity and biochemical factors were likely to be important mechanisms of resistance. However, in the 24th-u generation, after fipronil selection had ceased for 9 generations, only a minimal decrease in RR was observed after treatment with synergists, suggesting that the double mutation (R300Q and A302S) was the primary factor in determining fipronil resistance. The detailed data for synergistic effects have been summarized in Table 1.

### Mutation detection in field populations

**Figure 3:**
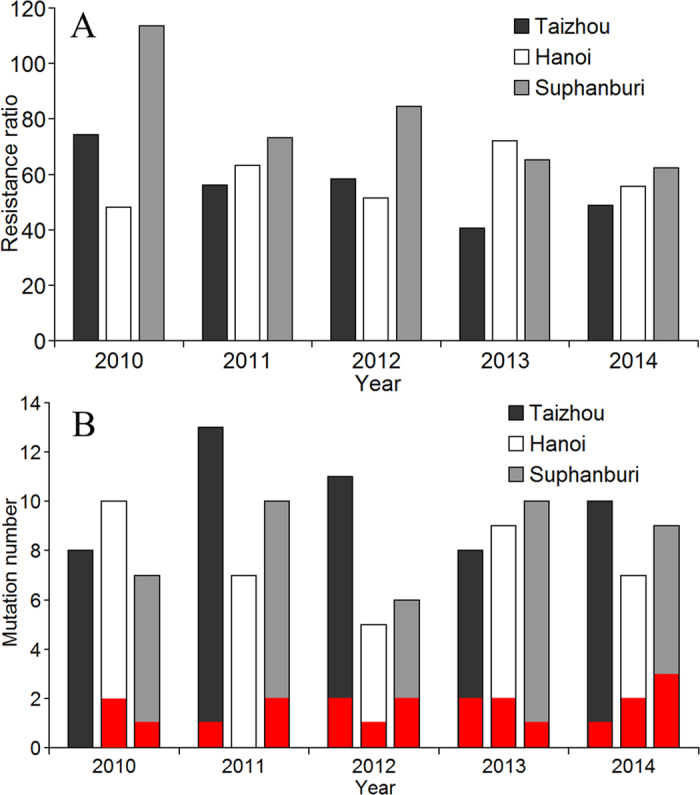
Fipronil resistance monitoring and mutation detections in *Nilaparvata lugens* field populations from Taizhou (China), Hanoi (Vietnam) and Suphanburi (Thailand) in five consecutive years from 2010 to 2014. (**A**) Fipronil resistance ratios in three field populations in five years. (**B**) Mutations detected in field populations. Columns (black, white and grey) indicate the number of individuals with A302S mutation among 300 tested individuals in each population. In each column, the red bar indicates the number of individuals with the double mutation (R300Q/A302S). No individuals with only R300Q mutation were detected.

In order to determine the relative frequency of the two RDL mutations in field populations of *N. lugens*, insects were collected in 2012 from 12 different locations and from three different countries (China, Vietnam and Thailand) (Table 2). The A302S mutation was detected in all field populations at a frequency of 1–5% (Table 2). The R300Q mutation was found in some field populations but always at a lower frequency than A302S in the corresponding population. Significantly, the R300Q mutation was always detected in combination with the A302S mutation. No individuals were found, in any of the populations examined, which contained the R300Q mutation in the absence of A302S. These findings indicated that R300Q could be a harmful or lethal mutation and also suggested that the A302S mutation might be able to compensate for the fitness cost associated with R300Q. In addition, field populations were collected from Taizhou (China), Hanoi (Vietnam) and Suphanburi (Thailand) in five consecutive years from 2010 to 2014. The bioassay results showed that fipronil resistance remained above 40-fold, even in field populations from Taizhou after the use of fipronil was prohibited on rice planthoppers in China in 2010. The single mutation A302S and double mutation R300Q/A302S were detected in all field populations, although the frequency of the double mutation was low (Fig. 3). These results indicated that fipronil resistance and related target mutations were widely distributed in field populations, but the low frequency indicated that the target mutations were not the dominant mechanism for fipronil resistance in the field populations that we have examined.

### Fitness cost determination

Life tables for several generations were constructed to analyze the influence of fipronil resistances on fitness costs (Table 3). During selection, the relative fitness decreased to 0.74 for the 7th generation and close to 0.6 for the 17th and 24th generations. In the 24th-u generation (after fipronil selection had ceased for 9 generations), the double mutation remained at a frequency of 100% (Fig. 1B) but there was no significant effect on fitness in comparison to the 1st generation (Table 3). It seems likely therefore that effects on fitness seen in the 17th and 24th generations, where the double mutation was also present at a frequency of 100%, were a consequence of the involvement of detoxification mechanisms. However, it has not been possible within the scope of this study to determine which detoxification mechanisms may be involved. Taken together, the data for mutation detection, synergistic effects and fitness cost analysis, it seems reasonable to conclude that the double mutation R300Q/A302S, rather than a single mutation, is required to provide persistent and high levels of resistance to fipronil in *N. lugens*.

### Recombinant RDL expressed in *Xenopus* oocytes

**Figure 4:**
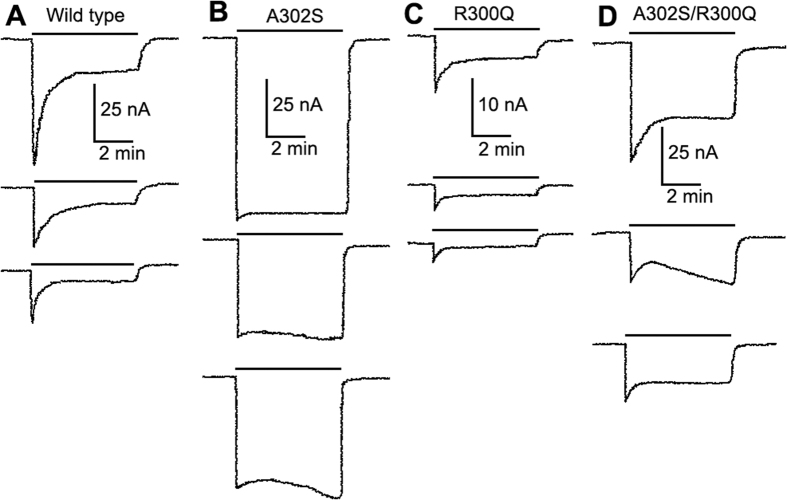
Effects of fipronil on GABA-activated currents in *Xenopus* oocytes expressing wild-type and mutant RDL receptors. Representative traces are illustrated from wild-type RDL. (**A**) A302S (**B**), R300Q (**C**) and R300Q/A302S (**D**). In each column, the top trace is a response to an *EC*_30_ concentration of GABA, the middle trace is a response to the same concentration of GABA co-applied with 0.1 μM fipronil and the bottom trace is a response to *EC*_30_ of GABA after a 15-min wash. Because the four RDL variants have different sensitivity to activation by GABA, an equipotent concentration of GABA (*EC*_30_) was used in each case (25, 10, 206 and 33 μM, respectively).

To examine the effects of the R300Q and A302S mutations on the electrophysiological properties of RDL, cDNA encoding *N. lugens* RDL was expressed in *Xenopus* oocytes. Bath application of GABA resulted in inward currents in cells expressing wild-type RDL. Similarly, functional responses were observed with RDL containing the double mutation (R300Q and A302S) or containing either of these mutations singly (Fig. 4). On wild-type and mutant R300Q RDL, the induced currents displayed rapid desensitization (Fig. 4A,C). In contrast, on mutants with the A302S mutation or with the double mutation, desensitization was less pronounced (Fig. 4B,D). When co-applied with GABA, fipronil inhibited the induced current by GABA application on all receptors and little or no recovery from inhibition was observed after a 15-minute wash (Fig. 4).

**Figure 5:**
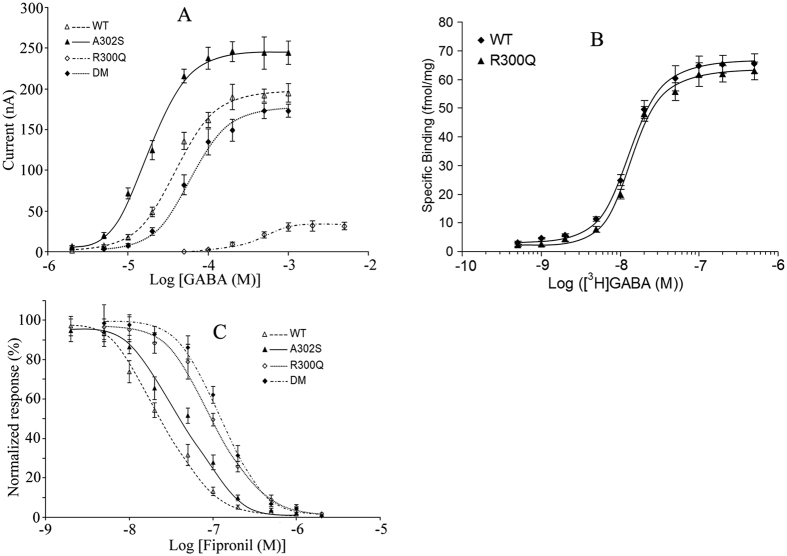
Characterization of wild-type and mutant RDL receptors expressed in *Xenopus* oocytes. Data are presented, from wild-type RDL and RDL containing single mutations (A302S or R300Q) or a double mutation (DM; A302S and R300Q), illustrating agonist dose-response curves to a range of concentrations of GABA (**A**), radioligand binding studies with [^3^H]-GABA (**B**) and dose-response curves illustrating the inhibition by fipronil of responses to GABA (**C**). Antagonist effects to a range of concentrations of fipronil were examined on wild-type and mutated RDL. Because the four RDL variants have different sensitivity to activation by GABA, an equipotent concentration of GABA (*EC*_30_) was used (25, 10, 206 and 33 μM for wild-type RDL, A302S mutant, R300Q mutant and A302S/R300Q mutant, respectively). Data are means of at least five independent experiments.

GABA dose-response curves gave a mean *EC*_50_ of 38 μM on wild-type RDL receptors (Fig. 5A). The potency of GABA on A302S mutant (*EC*_50_ = 19 μM) was 2.0 times higher than that on the wild-type receptor. Surprisingly, GABA potency on R300Q mutant (*EC*_50_ = 413 μM) was 10.9 times lower than that on wild-type receptors. When the double mutation (R300Q and A302S) was introduced, the estimated *EC*_50_ value (54 μM) was similar to that on wild-type receptors. The Hill coefficients did not differ significantly between the wild-type or mutated receptors (Table 4).

Fipronil showed significant inhibition on GABA potency for the wild-type receptors, with *IC*_50_ of 20 nM (Fig. 5C). Both A302S and R300Q resulted in a significant shift of the inhibition curves to the right, with estimated *IC*_50_ values of 45 and 96 nM respectively. In addition, the double mutation shifted the curve further to the right, with an *IC*_50_ value of 125 nM (Table 5).

### Radioligand binding to oocyte membrane proteins with different RDL receptors

[^3^H]-GABA binding to oocyte membrane proteins expressing wild-type or mutated (R300Q) RDL was performed to analyze the influence of R300Q on GABA binding (Fig. 5B). No significant differences were found in either *B*_max_ (67.4 ± 4.7 fmol/mg and 65.1 ± 6.2 fmol/mg for wild-type and R300Q, respectively) or *K*_d_ (12.3 ± 2.1 nM and 13.4 ± 2.7 nM for wild-type and R300Q, respectively). These results indicate that the R300Q mutation exerts its influence on the functional properties of RDL, rather than by influencing the binding of GABA to the receptor.

## Discussion

Resistance, whether to pesticides, antibiotics or antiviral drugs, is a widespread problem that can result in substantial agricultural, medical and economic costs. Although resistance to xenobiotics can occur as a consequence of enhanced metabolism or detoxification, an important mechanism that can confer high levels of resistance is the mutation of a target site protein^1^,^24^,^25^. Changes caused by mutations to a target protein are often deleterious to the survival of an organism because they impair the ability of the protein to function normally^4^,^26^,^27^,^28^,^29^, particularly since there is often strong evolutionary pressure to conserve protein structure^30^,^31^. Consequently, organisms subjected to agents such as pesticides, antibiotics or antiviral drugs are subjected to conflicting selective pressures. A potential advantage can be gained by mutations conferring resistance to xenobiotics, but there is an opposing need to conserve normal protein function. One approach to resolving this dilemma may be the accumulation of two mutations that act synergistically to confer resistance but minimize fitness cost to the organism.

There is extensive evidence for such compensatory mechanisms operating in viruses and bacteria^24^,^32^,^33^, whereby a compensatory mutation can mask the deleterious effects caused by other mutations^34^,^35^. A number of compensatory mechanisms are possible, including both intragenic and intergenic mutations^24^. There are also reports of compensatory resistance mechanisms in insects, such as the duplication of a wild-type copy of a target gene to compensate for fitness cost caused by a mutation in the same target, or by a mutation in one target compensating for a fitness cost associated with a mutation in another^5^,^9^,^10^,^36^. However, these examples of insecticide resistance can be considered as intergenic compensatory mechanisms. In contrast, whereas intragenic mutations are a common compensatory mechanism of resistance in viruses or bacteria, they appear to be less common in insects. Multiple mutations, associated with insecticide resistance within a single gene, have been reported but, in general, have not been shown to result in compensatory mechanisms whereby higher levels of resistance are associated with lower fitness cost.

Previous studies have reported the existence of resistance-associated mutations in RDL that can coexist with A302S. A mutation (V327I) has been reported to coexist with A296S (analogous to position 302 in *D*. *melanogaster*) in dieldrin-resistant *Anopheles funestus*^37^. Similarly, the mutations T350M and A301G (analogous to position 302 in *D*. *melanogaster*) have been identified in fipronil resistant *Drosophila simulans*^38^ and in the mosquito *Anopheles gambiae*^39^. In addition, it has been suggested that the R340Q mutation, in combination with A2’N (corresponding to position 302 in *D. melanogaster*) is associated with fipronil resistance in the white-backed planthopper *Sogatella furcifera*^40^. However, in contrast to the findings reported here, these studies do not appear to provide direct evidence for an interaction between two mutations, by which one mutation is able to compensate for the deleterious effects on fitness cost of a second mutation. Nevertheless, some authors have highlighted this as a plausible mechanism by which the fitness cost of resistance-associated mutations might be reduced^39^,^41^.

Although mutations at the A302 position in RDL have been identified as conferring high levels of resistance to dieldrin and aldrin in several insect species, the role of such mutations in conferring resistance to fipronil appears to be inconsistent, with effects varying from relatively minor^42^,^43^ to more substantial^44^,^45^,^46^,^47^. As a consequence, it is difficult to assess the likely contribution of A302S mutation to fipronil resistance in insects. In the present study, we have identified a population of insects containing only the A302S mutation (e.g. in the 7th generation) that has relatively low fipronil resistance (with RR of 23 in the absence of synergists and of 5 in the presence). However, insects containing the double mutation (R300Q and A302S) have much higher levels of resistance (with RR of 235 in the absence of synergists and of 100 in the presence, e.g. in the 17th generation). An interesting aspect of our findings is that the R300Q mutation was never detected in the absence of A302S mutation in individuals from either laboratory-selected or field populations. A likely explanation for this is provided by studies of the R300Q mutation in recombinant RDL receptors, where the mutation had a profound effect on the normal functioning of the receptor in response to the endogenous agonist GABA. Importantly, however, the deleterious effects of R300Q can be compensated for by the A302S mutation. Our electrophysiological studies help to support the conclusion that the R300Q mutation is responsible for increased fipronil resistance, whereas the A302S mutation provides a compensatory mechanism to reduce the otherwise deleterious effects caused by R300Q. Although the double mutation confers high levels of resistance to fipronil in a laboratory-selected strain of *N. lugans* and is also present in field populations, our finding suggest that it is not currently the dominant mechanism for fipronil resistance in field populations that we have studied, where other resistance mechanisms, including detoxification may be important.

Typically, where a resistance-associated mutation results in a significant fitness cost, the mutation may disappear in insect populations after removal of the selection pressure of the insecticide. In contrast, advantageous mutations might be sustained after removing insecticide application. Examples include the N1575Y and V1016I mutations in voltage-gated sodium channels from *An. gambiae*^48^, and *Ae. aegypti*^49^ and the G265A mutation in acetylcholinesterases of *D. melanogaster*^50^,^51^. In the absence of A302S, the R300Q mutation, identified in the present study, would be expected to have extremely deleterious effects on GABA receptor function. However, the co-occurrence of the R300Q and A302S mutations would appear to provide a solution to the otherwise deleterious effects of R300Q and provides a mechanism whereby insects can achieve high and persistent levels of resistance to insecticides.

In conclusion, two resistance-associated mutations (R300Q and A302S) have been identified in the GABA receptor RDL that exert synergistic and compensatory effects. Whereas the A302S mutation has been described previously, we have identified a second resistance-associated RDL mutation (R300Q) that, in combination with A302S, is associated with much higher levels of resistance to fipronil. The R300Q mutation has not been detected in the absence of A302S in either laboratory-selected or field populations, a finding that we attribute to the high fitness cost that is associated with this mutation when it occurs in the absence of A302S. These findings may have important implications for the spread of insecticide resistance in insect populations.

## Materials and Methods

### Brown Planthopper (*N. lugens*)

The susceptible (S) strain of *N. lugens* is a laboratory strain, obtained from Jiangsu Academy of Agricultural Sciences (Nanjing, China) in April 2000, which had been collected before fipronil application and reared in a greenhouse for >10 years. Bioassay and resistance selection were performed using the rice-stem dipping method with third instar nymph^52^. Fipronil was dissolved into acetone and then diluted into six concentrations with water. Bioassays (to determine *LC*_50_ values) were performed by applying each concentration of fipronil separately to the 4th instar nymphs in each generation or population. Laboratory resistance selection was performed on an insect population that was collected from Chainat, Thailand, in July 2009. The selection was performed in a repeated cycle including two generations, in which the first generation selection was with a 2 × *LC*_99_ concentration and the second with *LC*_50_ concentration. After the 16th generation, the population was divided into two sub-populations. One sub-population was continuously selected in the repeated cycle for four generations and with *LC*_50_ concentrations following the 20th generation. The other sub-population was reared under the same condition without insecticide selection. In July–August from 2010 to 2014, field populations were collected from Taizhou (China), Hanoi (Vietnam) and Suphanburi (Thailand) for the bioassay and mutation detections. In July–August 2012, nine field populations were also collected from China (Guilin, Jiujiang and Nanjing), Vietnam (Ho Chi Minh, Tien Giang and Dong Thap) and Thailand (Ang Thong, Chainat and Chiengmai) for mutation detections. In examination of synergistic effects, 6 μg of synergists (2 μg for each of triphenyl phosphate, diethyl maleate and piperonyl butoxide) in 0.08 μl acetone was delivered onto the prothorax notum of the 4th instar nymph 1 h before the bioassay^53^.

### Life table construction

Life tables were constructed as described previously^54^. One hundred neonates were collected randomly from a population and reared for a generation at 25 ± 1 °C and 16/8 h light/dark period. When insects had developed into the 3rd instars, the hoppers were transferred to fresh rearing cages and assessed for survival rate from neonate to the 3rd instar (Sr1). When insects had developed into the 5th instars, the hoppers were transferred to fresh rearing cages again and assessed for survival rate from the 3rd to 5th instar (Sr2). The emerged males and females were thereafter collected every day and paired into ‘families’ (approximately ten families of one female plus one male for each replication, in total about 30 for each population), which were reared separately in glass tubes. At the same time, the emergence rate (Er) and female ratio (Fr) were recorded. When the neonates of the new generation appeared, the families were checked every two days and the neonates were counted and removed until the female died. If the female or male in one family died within 4 days of pairing, this family was excluded from the study and not counted in the total family number. The food rice shoots were then checked thoroughly and the number of unhatched eggs was recorded. The females which had not produced any neonates were considered to have failed in copulation, and the copulation rate (Cr) was accounted accordingly. The fecundity (Fd) was recorded as the average number of eggs produced by copulated females, and the hatchability (Ha) was recorded as (all neonates)/(all neonates plus all unhatched eggs). The experiments were carried out with three replications. The population trend index (*I*) and relative fitness were calculated as follows:


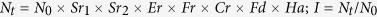



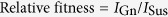


### RT-PCR

Total RNA was extracted from a single individual of the adult *N. lugens* using Trizol (Invitrogen, USA). Synthesis of first-strand cDNAs and RT-PCR were performed as described previously^55^. PCR reactions were performed with 0.20 mM dNTP, 1.00 μM gene specific primers (forward primer: GTTCATTCGGATACACC ACTCTGG; reverse primer: CCTTAGGATCGTGAACCTTGAAG) and 1.25 U *Pfu* DNA polymerase (Promega, Madison, WI, USA) in a total volume of 50 μL. Thermal cycling conditions were 95 °C for 5 min followed by 35 cycles of 95 °C for 40 s, 60 °C for 40 s and 72 °C for 1 min, with a final extension at 72 °C for 8 min. PCR products were purified by electrophoresis in 1% agarose gel and examined by nucleotide sequencing (Invitrogen, USA). In each generation during selection, 100 individuals were sequenced. In each field population, 300 individuals were tested. Oligonucleotide primers were designed based on the *N. lugens* RDL nucleotide sequence (Genbank accession number: KC841916).

### Plasmids and *in vitro* transcription

Wild-type and mutated *N. lugens* RDL were subcloned into *Eco*RI and *Xba*I sites of pGH19 as described previously^56^. All plasmid constructs were verified by nucleotide sequencing. Plasmid pGH19 constructs encoding *N. lugens* RDL was linearized with *Not*I and capped RNA transcripts were synthesized by the SP6 polymerase, using the mMessage mMachine transcription kit (Ambion, Austin, USA). Transcripts were recovered by precipitation with propan-2-ol, dissolved in nuclease free water at a final concentration of 0.5 mg/mL and stored at −80 °C prior to use.

### Oocyte preparation and electrophysiology

The *Xenopus* were kindly provided by Stem Cell Bank, Chinese Academy of Sciences. The methods for *Xenopus* oocyte preparation were as described previously by Le Goff *et al*.^57^ with some minor modifications^56^ and have been approved by the Ethics Committee of Nanjing Agricultural University. cRNA (50 ng) was injected into each oocyte. The oocytes were incubated for approximately 60 h at 18 °C in standard oocyte saline (SOS, NaCl 100 mM, KCl 2 mM, CaCl_2_ 1.8 mM, MgCl_2_ 1 mM, HEPES 5 mM, pH 7.5) containing 5% heat-inactivated horse serum (Invitrogen, Paisley, UK) and then stored at 4 °C. Electrophysiological recordings were performed using a two-electrode voltage clamp amplifier (Multiclamp 700B Amplifier, Axon Instruments, USA) as previously described^56^. Membrane currents were recorded at a holding potential (Vh) of −60 mV and displayed on a dual channel chart recorder (Warner Instruments, USA). Dose-response curves for GABA were constructed for susceptible and mutant RDL receptors using oocytes that yielded stable responses to GABA. Fipronil (98%, Rhone-Poulenc S.A., France) was first dissolved in dimethylsulphoxide (DMSO). The stock solutions were then diluted in SOS to the required final concentration. Fipronil was added to the superfusate at the end of GABA application and continuously applied thereafter. At 15 min, GABA (*EC*_30_) was co-applied with the antagonist for 4 min. The currents were measured at peak. Following the second test application of GABA, the preparation was washed with SOS for 15 min and recovery was assessed using a last application of GABA. To construct dose-response curves, typically nine concentrations were applied to a single oocyte and replicate experiments were performed on at least five different oocytes.

### Oocyte membrane protein extraction and radioligand binding

Membrane proteins from *Xenopus* oocyte were prepared and [^3^H]-GABA binding was performed as described previously^58^. Oocytes were homogenized in 2 ml of extraction buffer [pH 7.2, 0.32 mM sucrose, 100 μM EDTA, 1% proteinase inhibitor mixture I (Sigma)]. The homogenate was centrifuged at 1,000 × g for 30 min. The resultant supernatant was filtered through four layers of cheesecloth and centrifuged at 30,000 × g for 60 min. The pellet was resuspended in the incubation buffer (pH 7.4, 0.05 mM Tris, 0.12 mM NaCl, 100 μM EDTA). [^3^H]-GABA (33.4 Ci/mmol) was purchased from Perkin-Elmer Life and Analytical Sciences (Waltham, MA). Samples were assayed by filtration onto Whatman GF/B filters presoaked in 0.5% polyethylenimine, followed by rapid washing by using a cell harvester (Brandel, Bethesda, MD). Amounts of total protein were determined by a Bio-Rad DC protein assay using BSA standards.

### Data analysis

Pooled data are presented as means ± SEM of at least 5 independent experiments. *EC*_50_, *IC*_50_ values and slope factors (Hill coefficients) were calculated by a non-linear least-squares curve fitting program (Prism 5.0, GraphPad Software, USA). Statistical significance was determined by one-way ANOVA, together with Fisher’s least significant difference post-hoc test for pair wise comparisons. Data were considered to be significant if *p* < 0.05.

## Additional Information

**How to cite this article**: Zhang, Y. *et al*. Synergistic and compensatory effects of two point mutations conferring target-site resistance to fipronil in the insect GABA receptor RDL. *Sci. Rep.* **6**, 32335; doi: 10.1038/srep32335 (2016).

## Figures and Tables

**Table 1:** Fipronil toxicity and synergistic effects of synergists in the susceptible strain and two specific generations during selection.

Population	Treatment	Slope	LC_50_ (mg/kg)	95% confidence limit	Resistance ratio
S strain	Fipronil	2.74 ± 0.31	0.153	0.144–0.167	1.00
	+synergists	2.35 ± 0.44	0.137	0.125–0.162	0.90
7th generation	Fipronil	1.72 ± 0.29	3.548	3.024–4.331	23.19
	+synergists	1.46 ± 0.37	0.717	0.635–0.792	4.68
17th generation	Fipronil	1.95 ± 0.22	36.016	31.512–42.718	235.40
	+synergists	1.70 ± 0.32	15.261	12.404–18.383	99.75
24th generation	Fipronil	2.13 ± 0.19	35.068	31.447–39.512	229.20
	+synergists	1.86 ± 0.36	14.865	11.316–19.260	97.16
24th-u generation	Fipronil	2.27 ± 0.31	16.039	13.209–19.874	104.83
	+synergists	1.95 ± 0.33	12.661	10.002–16.461	82.75

**Table 2:** Mutation detection in field populations.

Country	Collection site	A302S	R300Q	DM
China	Guilin	4	0	0
	Jiujiang	6	1	1
	Taizhou	11	2	2
	Nanjing	7	2	2
Vietnam	Ho Chi Minh	12	3	3
	Hanoi	5	1	1
	Tien Giang	3	0	0
	Dong Thap	8	1	1
Thailand	Suphanburi	6	2	2
	Ang Thong	8	1	1
	Chainat	10	2	2
	Chiengmai	4	0	0

In each population, 300 individuals were used to detect A302S and R300Q mutations. DM, double mutation. The same numbers of R300Q and DM in each population showed all R300Q mutations were detected in individuals with A302S mutation.

**Table 3:** Life tables for different populations of *Nilaparvata lugens.*

Parameter	1st generation	7th generation	17th generation	24th generation	24th-u generation
Neonate number	100	100	100	100	100
Survival rate from neon ate to 3rd instar (%)	90.7 ± 2.4a	80.8 ± 3.5b	77.4 ± 3.8b	78.9 ± 4.1b	88.3 ± 4.9a
Survival rate from 3rd to 5th instar (%)	92.3 ± 4.1a	91.6 ± 4.7a	90.2 ± 6.2a	91.4 ± 7.1a	92.9 ± 5.4a
Emergence rate (%)	91.1 ± 3.7a	82.7 ± 4.1b	81.6 ± 3.6b	80.2 ± 4.3b	92.0 ± 4.4a
Female ratio (%)	49.2 ± 2.9a	48.1 ± 3.8a	48.9 ± 4.5a	47.8 ± 3.9a	48.8 ± 3.7a
Copulation rate (%)	86.3 ± 5.3a	88.4 ± 6.2a	84.9 ± 4.8a	83.9 ± 6.7a	87.9 ± 5.1a
Fecundity (eggs per female)	354.8 ± 28.9a	328.5 ± 36.4ab	307.9 ± 29.3b	305.5 ± 31.8b	369.7 ± 38.2a
Hatchability (%)	87.3 ± 4.6a	86.4 ± 5.1a	86.2 ± 6.0a	85.8 ± 4.8a	86.2 ± 5.5a
N, predicted number of offspring	10030.0	7386.8	6277.3	6079.8	10316.4
I, population trend index	100.3	73.9	62.8	60.8	103.2
Relative fitness	1.00	0.74	0.63	0.61	1.03

Different values in the same row showed the significant difference at 0.05 level.

**Table 4:** Influence of mutations on GABA potency.

RDL	*I*max (nA)	*EC*_50_ (μM)	Hill coefficient
Wild-type	195.56 ± 27.43b	37.72 ± 5.40b	1.74 ± 0.11a
A302S	244.08 ± 35.21a	18.84 ± 3.37a	1.78 ± 0.14a
R300Q	31.94 ± 4.39c	413.44 ± 67.15d	1.68 ± 0.15a
A302S/R300Q	173.73 ± 22.26b	54.08 ± 8.21c	1.64 ± 0.13a

Values are means ± SEM for at least 5 experiments. Different letters in the same column showed the significant differences at 0.05 level.

**Table 5:** Influence of mutations on fipronil sensitivity.

RDL	*IC*_50_(nM)	n*
Wild-type	19.81 ± 3.31a	6
A302S	45.47 ± 7.05b	6
R300Q	96.36 ± 11.27c	8
A302S/R300Q	124.75 ± 16.03d	9

Different letters in the same column showed the significant differences at 0.05 level. Values are means ± SEM for experiment repeats as indicated by * in the table.
